# Supplementary material for: WordCommentsAnalyzer: A windows software tool for qualitative research
Source: F1000Res. 2018 Sep 4;7:536. Originally published 2018 May 3. [Version 2] doi: 10.12688/f1000research.14819.2 (PMC6137411; doi:10.12688/f1000research.14819.2)
Supplement: Supplementary file 3 [file f1000research-7-17614-s0002.tgz › b2a79496-4ada-47c4-9682-a99ce867836d.zip!/Eight Iranian Health Journals/Archives of Rehabilitation_70.docx]

© Archives of Rehabilitation. This work is licensed under CC BY-NC 4.0 (https://creativecommons.org/licenses/by-nc/4.0/).

Volume & Issue: دوره 2 - شماره 1 و 2

Title: بررسی شیوع اختلالات شنوایی در بین کودکان 3 تا 6 سال مهد کودکهای تحت پوشش سازمان بهزیستی استان تهران

http://rehabilitationj.uswr.ac.ir/article-1-532-fa.html

هدف: نقص شنوایی به معنای عملکرد غیرطبیعی یا کاهش عملکرد شنوایی به دلیل وجود نوعی اختلال شنوایی است. هدف هر برنامه غربالگری شنوایی در سنین پیش دبستانی و دبستانی، شناسایی دقیق آن دسته از کودکانی است که شنوایی آنها به علت وجود نوعی ضایعه انتقالی یا حسی-عصبی آسیب دیده است. اگر چه برخی از اختلالات گوش میانی (مانند ترشح گوش میانی) ممکن است کم شنوایی قابل توجهی ایجاد نکنند، شناسایی آنها بخشی از برنامه غربالگری شنوایی است، چراکه می توانند بر توانایی یادگیری کودک تأثیر گذار باشند. روش بررسی: مطالعه توصیفی حاضر به صورت مقطعی، از فروردین تا خرداد سال 1380 روی 577 کودک (278 دختر و 299 پسر) انجام شد. این کودکان به صورت تصادفی، از بین کودکان مهدهای کودک تحت پوشش سازمان بهزیستی استان تهران انتخاب شدند. پس از تکمیل برگه آگاهی از وجود کم‌شنوایی توسط والدین، معاینه اتوسکوپی، غربالگری تون–خالص و غربالگری ایمیتانس صورت می گرفت. یافته‌ها: در این بررسی، %12/58 اختلال مجرای گوش خارجی، %23/7 اختلال پرده گوش، %34/3 تیمپانوگرام ناهنجار، %35 عدم وجود رفلکس صوتی و %12/6 درصد کم‌شنوایی شامل %9/7 درصد کم‌شنوایی دوطرفه و %2/9 کم شنوایی یک طرفه (%11/6 درصد کم شنوایی انتقالی، %0/64 کم شنوایی حسی-عصبی و %0/36 کم شنوایی آمیخته) مشاهده شد. 1/34 درصد کودکان نیازمند اقدامات درمانی و یک نفر (%0/2) از کودکان ، نیازمند خدمات توانبخشی بودند و میزان آگاهی والدین (%1/73) و مربیان (%2/07) از وجود اختلال شنوایی کودک در سطح پایینی قرار داشت. همچنین شیوع بیشتر اختلال شنوایی در گوش چپ و تفاوت معنی دار بین دو گوش (P<0/05) در کلیه موارد بررسی (به جز وضعیت رفلکس صوتی) مشاهده شد. نتیجه‌گیری: با توجه به درصد بالای نیاز به اقدامات درمانی و اثرات سوء اختلالات شنوایی بر یادگیری کودکان در سنین پایین و همچنین اهمیت شناسایی و مداخله زود هنگام کم شنوایی در برنامه های توانبخشی شنوایی، آگاهی رسانی و آموزش به افراد جامعه درباره اثرات کم‌شنوایی و نحوه پیشگیری و شناسایی آن از اهمیت بسزایی برخوردار است.

Title: شناخت عوامل موثر در استئوآرتریت زانو و برآورد شیوع آنها در مراجعان به درمانگاههای ارتوپدی تهران

http://rehabilitationj.uswr.ac.ir/article-1-533-fa.html

هدف: هدف از این مطالعه شناسایی عوامل مؤثر در آستئوآرتریت زانو برآورد شیوع آن در زانوها بوده است. روش بررسی: نوشتار فوق حاصل مطالعه ای گذشته نگر است و روی پرونده های 176 نفر از مراجعه کنندگانی که از درد زانو شکایت داشته و طی آبانماه 1377 لغایت مردادماه 1378 به درمانگاه های ارتوپدی تهران مراجعه کرده اند و تشخیص استئوآرتریت برای آنها داده شده، انجام گرفته است. انتخاب پرونده ها به طور تصادفی بوده است. یافته ‌ها: شیوع استئوآرتریت در «فقط زانوی چپ» برابر %22/9، در «فقط زانوی راست» 9 برابر %39/4 و در هر «دو زانو» %37/6 است. متغیر جنس بر شیوع این بیماری اثر معنی داری دارد (P<0/002)  به طوری که شیوع در زنان بیش از مردان دیده می شود. هر چند به‌طور کلی شیوع این بیماری در شهر بیشتر از روستاست، ولی این اختلاف معنی دار نیست و محل سکونت اثر معنی داری بر این بیماری نشان نمی‌دهد (P<0/1471). با افزایش سن، شیوع استئوآرتریت زانو افزایش می‌یابد (P<0/001). به طور کلی، شغل افراد بر استئوآرتریت زانو مؤثر است، به طوری که، شیوع این بیماری در افرادی که کارهای یدی دارند، بیشتر از کسانی است که کار فکری انجام می دهند. در بین عوامل تشدید درد، هم در مجموع، هم در «هر دو زانو» و هم در «فقط زانوی راست»، بیشترین تأثیر را «نشستن چهار زانو» و کمترین اثر را «پیاده روی کوتاه» داراست، در حالی که «پیاده روی طولانی» در «فقط زانوی چپ» بیشترین اثر و «در حال استراحت» کمترین اثر را نشان نی دهد. «استراحت کامل» هم در مجموع، هم در هر دو زانوی راست، به طور متوسط %40 باعث بهبودی درد می شود. در حالی که هم در مجموع، هم در زانوی راست و هم در هر دو زانو، «نشستن روی صندلی و مبل» کمترین اثر را در بهبودی درد دارند، ولی در «فقط زانوی چپ»، «دراز کردن پا» کمترین اثر را در بهبودی درد داشته است. نتیجه‌گیری: عوامل فیزیکال شامل تغییر شکل ظاهری مفصل در حال ایستادن، دامنه حرکات زانو و پارگی در منیسک‌ها بر این بیماری اثر معنی داری دارند، همچنین عواملی که از طریق مشاهدات رادیولوژیک مشاهده شده اند نظیر لاغری در مفصل، اسکلروزه شدن استخوان زیر غضروف، تشکیل کیست، تشکیل استئوفیت و غیر طبیعی بودن زائو از نظر بیومکانیکی بر استئوآرتریت زانو اثر معنی دار دارند. متغیرهای قد، وزن، ضربه دیدن زانو، سابقه عفونت آن، استفاده از صندلی و مبل، درمان های دارویی، نوع سیستم بهداشتی، ورزش کردن، درمان های غیر دارویی از جمله دیگر عوامل مؤثر بر این بیماری هستند.

Title: درمان و توانبخشی عصبی-عضلانی در بیماران مبتلا به فلج عصب صورتی

http://rehabilitationj.uswr.ac.ir/article-1-534-fa.html

هدف: با وجود مواردی از فلج فاسیال و وجود گزارشات متعدد درباره درمان این بیماران و به منظور نقش تأثیر روش جراحی و توانبخشی بر روی بیماران مبتلا به فلج فاسیال، مطالعه روی بیمارانی که مبتلا به فلج فاسیال بوده و به بیمارستان 15 خرداد و بیمارستانهای خصوصی تهران در سال 75-1370 مراجعه کرده بودند صورت گرفته است. روش بررسی: تحقیق به روش کارآزمایی بالینی روی تعداد 80 بیمار صورت گرفت. این بیماران به دو گروه عمل نشده به تعداد 22 بیمار و گروه عمل شده که محتوی 58 بیمار بود تقسیم شدند . بیماران حداقل پنج سال پیگیری شدند و از نظر بسته شدن پلک چشم و حرکت لبها و قرینه بودن حرکات عضلات صورت مورد بررسی و ارزیابی قرار گرفتند. یافته‌ها: از تعداد هشتاد بیمار 22 بیماری که جزء گروه عمل نشده بودند با درمان داروئی و فیزیوتراپی به صورت تحریکات الکتریکی عضلات صورت درمان شدند . تعداد 58 بیمار که تحت عمل جراحی قرار گرفتند 38 بیمار فلج فاسیال متعاقب تروما و بیست بیمار فلج فاسیال در اثر فلج بلز و بیماری مادرزادی داشتند . درمان های  جراحی انجام شده عبارت بوده از پیوند مستقیم دو سر قطع شده عصب، گرافت عصب بین دو سر قطع شده ، نوروتیزاسیون از طرف سالم برای طرف فلج، نوروتیزاسیون با عصب هیپوگلوس یا عصب شوکی طرف معیوب بوده است. استفاده از عضلات جانشین مثل عضله تمپورال و گراسیلیس از روش های دیگر درمانی بوده است. نتیجه‌گیری: تمام بیمارانی که در اثر فلج بلز و یا متعاقب عمل جراحی تومور مغزی دچار فلج فاسیال شده اند باید در ابتدا تحت درمان پردنیزولون و تحریکات الکتریکی برای عضلات صورت قرار بگیرند و در صورتی که این درمان مؤثر نبود باید از روشهای جراحی ترمیمی استفاده شود. توصیه می شود که فلج فاسیال کهنه تحت عمل جراحی انتقال عضلات آزاد دو مرحله ای برای به دست آوردن حرکت قرینه عضلات صورت انجام بگیرد.

Title: پ‍‍ژوهشی پیرامون مشکلات روانی-اجتماعی نوجوانان «پدر معتاد»

http://rehabilitationj.uswr.ac.ir/article-1-535-fa.html

هدف: الکل و دیگر مواد مخدر بر زندگی نوجوانانی که والدین آنها به مصرف مواد مخدر وابسته باشند، تاثیر فراوان باقی می گذارد. این قبیل نوجوانان نه تنها بعداً در معرض خطرات ناشی از مصرف مواد مخدر هستند، بلکه از دیگر مشکلات روانی اجتماعی نیز رنج می برند . مطالعه حاضر با هدف شناسایی مشکلات روانی - اجتماعی (افت تحصیلی، اختلال در روابط اجتماعی، و بروز اضطراب) نوجوانان پدرمعتاد انجام گرفته است. روش بررسی:  از پرسشنامه 90 سوالی آزمون روانی–اجتماع (SCL-90)، پرسشنامه وضعیت تحصیلی و پرسشنامه حوادث زندگی استفاده شده است. این مطالعه از نوع مطالعات مورد–شاهدی است و در ابتدا، با مراجعه به مرکز بازپروری قرچک ورامین، تعدادی از معتادانی که دارای فرزند نوجوان 18-12 ساله (پسر و دختر) و معیارهای مورد نظر بودند، به صورت تصادفی انتخاب و سپس از بین فرزندان آنها 40 نفر به عنوان جامعه مورد تعیین شدند. به منظور مقایسه، تعداد 40 نفر نوجوان «پدر غیر معتاد» از فامیل و 40 نفر نوجوان «پدر غیرمعتاد» از همکلاسی‌ها که از نظر متغیرهای سن، جنس، سطح تحصیلی، تعداد فرزندان در خانواده سطح تحصیلات و شغل پدر و مادر همتا شده بودند، به عنوان گروه شاهد به طور تصادفی انتخاب و در آزمون شرکت داده شدند. یافته‌ها: در گروه مورد-شاهدی ما، افت تحصیلی، اضطراب و اختلال در روابط متقابل بیشتر است و بین اعتیاد پدر و مشکلات روانی - اجتماعی فرزندان نوجوان آنها، رابطه معنی داری وجود دارد .(P<0/05) نتیجه‌گیری: نتایج این پژوهش حاکی از تفاوت معنی دار در وضعیت تحصیلی، اختلال در روابط متقابل، و میزان اضطراب نوجوانان «پدر معتاد» و نوجوانان «پدر غیرمعتاد» است.

Title: بررسی وضعیت ستون فقرات و پارامترهای موثر بر کمردرد

http://rehabilitationj.uswr.ac.ir/article-1-536-fa.html

هدف: کمر درد یکی از شایعترین بیماریهاست که در تمام جوامع دیده می شود و امروزه در فیزوتراپی، برخی از ورزشهای ستون فقرات براساس کاهش یا افزایش قوس کمری تجویز می شود. لیکن، هر چند هنوز به درستی تأثیر قوس کمری بر کمردرد مشخص نشده است، یکی از عوامل مؤثر بر کمر درد شاید میزان قوس کمی باشد که تغییرات آن سبب ایجاد استرس بر ستون فقرات کمری می شود. بنابراین، هدف از این مقاله بررسی وضعیت ستون فقرات و تعیین رابطه بین قوس کمری و کمر درد در دانشجویان پسر یکی از خوابگاه های دانشگاه علوم پزشکی شهید بهشتی است. روش بررسی: سی و دو دانشجوی (5/2±21) ساله مورد بررسی قرار گرفتند. قوس کمری توسط خط کش قابل انعطاف، انحراف لگنی به وسیله شیب سنج، اسکولیوز توسط شاقول، افتادگی شانه با مقایسه قرینگی شانه ها و سابقه ورزشی با کمک پرسشنامه بررسی شد. برای تعیین reproducibility، اندازه گیری پارامترهای فوق برای هر فرد با فاصله زمانی حداقل پنج دقیقه تکرار شد. یافته‌ها: نتایج نشان داد که در جامعه مورد بررسی، %21/9 دارای اسکولیوز %34 دارای افتادگی شانه، %25 دارای صافی کف پا و %12/5 افراد دارای انحراف طرفی بودند. همچنین، %15/6 دانشجویان از کمر درد رنج می بردند. این نتایج، شیوع بالای ناهنجاری‌ها و دفورمیتی‌های ستون فقرات را نشان می‌دهد و لزوم آموزش عمومی در جهت تصحیح وضعیت (مخصوصاً برای افراد جوان) را می‌طلبد. لازم به ذکر است که ضریب همبستگی بین قوس کمری و انحراف لگنی، %43 بوده است که تأیید کننده نتایج برخی از پژوهشهای قبلی است. میانگین سن و نسبت قد و وزن تقریباً در افراد کمر دردی و سالم یکسان بود، لیکن درصد اسکولیوز، افتادگی شانه و صافی کف پا در افراد کمر دردی بیشتر از افراد سالم بوده است. همچنین، در افرادی که برنامه ورزشی های منظمی داشتند، شکایتی از کمر درد مشاهده نشد، لذا این مهم برتوجه بیشتر به ورزش تأکید دارد. نتیجه‌گیری: نتایج این تحقیق بین قوس کمری در افراد کمر دردی و سالم تفاوت معنی داری نشان نداد. از طرفی، میانگین قوس کمری در افراد دارای اسکولیوز بیشتر از افراد سالم بود، لیکن، در افراد کمر دردی که دارای افتادگی شانه و صافی کف پا بودند، قوس کمری کمتر از افراد سالم بوده است. این نتایج نشان می دهد که هیچ گونه ارتباط منطقی بین قوس کمری و کمر درد (یا بعضی از اختلالات که بر روی ستون فقرات تأثیر گذار است) وجود ندارد؛ بنابراین، تجویز ورزش‌های درمانی بر اساس کاهش یا افزایش قوسهای کمری، نیاز به بررسی و تحقیقات بیشتری دارد.

Title: بررسی قدرت گرفتن کروی و سه انگشتی در کودکان 4 تا 6 سال

http://rehabilitationj.uswr.ac.ir/article-1-537-fa.html

هدف: در این تحقیق، قدرت گرفتن کروی و سه انگشتی در 200 کودک راست دست و چپ دست از گروه سنی 6-4 سال با استفاده از الگوهای استاندارد تست قدرت که مورد تأیید انجمن امریکایی درمانگران دست و انجمن جراحان ارتوپدی است، مورد ارزیابی قرار گرفته است. این کودکان سالم و از مهد کودک های مناطق مختلف تهران انتخاب شده اند. سپس فرضیه های تحقیق ارزیابی شده است. روش بررسی: وسایل مورد استفاده مورد استفاده دراین تحقیق شامل مارتین ویگوریمتر، کولی، متر و ترازو بوده است. یافته‌ها: در این تحقیق نتایج زیر حاصل شد: اختلاف معنی داری بین میانگین های قدرت گرفتن کروی دست راست و چپ ، در دخترها و پسرها وجود ندارد، اما بین میانگین های قدرت گرفتن سه انگشتی دست راست و چپ در دخارها و پسرها ، اختلاف معنی دار وجود دارد. در کودکان راست دست اختلاف معنی داری بین میانگین های قدرت گرفتن کروی و سه انگشتی دست راست و چپ وجود دارد اما در کودکان چپ دست بین میانگین های قدرت گرفتن کروی و سه انگشتی دست راست و چپ اختلاف معنی داری وجود ندارد. نتیجه‌گیری: همبستگی مثبت و معنی داری بین قدرت گرفتن کروی و سه انگشتی با سن، وزن، قد، اندازه پهنای کف دست و اندازه طول انگشت میانی وجود دارد.

Title: بررسی میزان فراوانی شکایات و نیازهای توانبخشی در 150 سالمند مراجعه کننده به مرکز آموزشی پژوهشی و توانبخشی سالمندان

http://rehabilitationj.uswr.ac.ir/article-1-538-fa.html

هدف: در کشورهایی که طب سالمندی شناخته شده است ، تحقیقات فراوانی در رابطه با بهداشت و سلامت سالمندان انجام می‌شود. با توجه به اینکه طب سالمندی در کشور ما نیز در حال شناسایی است، این مطالعه با هدف بررسی وضعیت و فراوانی شکایات سالمندان و تعیین نیازهای توانبخشی آنان و مقایسه نتایج با مطالعات سالمندی در سایر کشورها انجام شده است. روش بررسی: این تحقیق یک مطالعه توصیفی است و روی 150 سالمند مراجعه کننده سرپایی به مرکز آموزشی، پژوهشی و توانبخشی سالمندان در سال 1378 انجام شده است و اطلاعات مورد نیاز با مطالعه پرونده این گروه جمع آوری شد. یافته‌ها: از 150 سالمند مراجعه کننده، %58 زن – %42 مرد بوده اند . حداقل سن 55 و حداکثر 100 سال بوده است. %17/3 بی‌سواد و %19/3 بالای دیپلم بوده اند. در بین زنان، %13 بیوه و در بین مردان %1/2 بیوه بوده اند. در این گروه 150 نفری 2 نفر زن مجرد ازدواج نکرده و یک نفر مرد ازدواج نکرده بوده است. شایع‌ترین شکایات سالمندان، عمدتاً بیماریهای قلب و ریه با %63/3 و بعد، شکایات اسکلتی عضلانی با %54/6، سپس شکایات خلقی و روانی %24/6 و بیماریهای مغز و اعصاب %18 بوده است. مجموعاً %35 سالمندان از برنامه‌های فیزیوتراپی، %13 از کاردرمانی و %2 از برنامه گفتار درمانی استفاده کرده اند. نتیجه‌گیری: با توجه به پایین بودن میزان ارائه خدمات توانبخشی در جامعه سالمندی کشور ما در مقایسه با کشورهایی که دارای طب سالمندی هستند، به نظر می رسد که رشته های توانبخشی و نقش آنها در افزایش کیفیت زندگی سالمندان، برای هر دو گروه سالمندان و پزشکان ما مورد غفلت قرار گرفته است، به‌ویژه، آشنایی بیشتر پزشکان با رشته های توانبخشی در طب سالمندی لازم به نظر می رسد.

Title: تأخیر تکلم در کودکان

http://rehabilitationj.uswr.ac.ir/article-1-539-fa.html

ادای اولین کلمات توسط کودک یکی از مهمترین شاخص های تکامل است. تکامل تکلم روند پیچیده ای دارد که هم از تکامل عصبی کودک و هم ارتباطات او (محیط) تأثیر می پذیرد. تکلم یک عمل تقلیدی نیست که در آن کودکان تنها آنچه را که می شنوند تکرار کنند، بلکه روند خلاقی است که از طریق آن کودکان به یک نظام قانونمند و نمادی دست می یابند. ایجاد تکلم از 5 بعد مهم قابل ارزیابی است: آواشناسی، معنی شناسی، قوانین تشکیل کلمه و حرف یا واژک شناسی، قوانین تشکیل جمله یا نحو و کاربردهای اجتماعی تکلم جهت برقراری ارتباط یا کاربرد شناسی. توانایی های درکی و بیانی کودک نیز بایستی ارزیابی شوند. این مقاله علاوه بر تعاریف فوق، به بحث در مورد اپیدیولوژی، علت شناسی و پاتوژنز، تظاهرات بالینی، چگونگی ارزیابی و ایجاد تشخیص زودرس، تشخیص افتراقی، درمان و توانبخشی و پیش آگهی تأخیر تکلم یا تکلم غیر طبیعی می پردازد. در این مقاله، همچنین بر مداخله زودرس در کودکانی که ثانویه به مشکلات رفتاری واضح شده اند و افرادی که مشکلات درازمدت کلامی آنها اثر سویی در تکامل اجتماعی شان داشته، تأکید می‌شود.

Title: زنان معلول معتاد

http://rehabilitationj.uswr.ac.ir/article-1-540-fa.html

طی قرنها، علی رغم کمکهای درمانی رسمی به زنان، فقط در چند دهه اخیر، مسأله ابتلای زنان به سوء مصرف مواد، مورد توجه قرار گرفته است. ماهیت و دلایل زیربنایی سوء مصرف مواد در زنان و مردان از یک شیوه مشابه پیروی نمی کند. و بالاخره تشخیص داده شد که نتایج تحقیقات مربوط به مردان را نمی توان به عنوان راه حل های مؤثر، در مورد زنان نیز به کار گرفت. در اینجا مسایلی مورد بحث قرار می گیرند که در کار با زنان معلول و مبتلا به سوء مصرف مواد می تواند ظهور کند،  زیرا، تجارب آنان از نظر بدنی، فرهنگی و اجتماعی، با مردان و سایر زنان متفاوت است. موضوع کلیدی بحث حاضر این است که زنان معلول و مبتلا به سوء مصرف مواد، در درجه اول و قبل از همه یک زن هستند که مسایل ویژه خود را دارند و در این زمینه، لازم است مشکلات آنان درک شود و در کار مشاوران، مددکاران اجتماعی و سایرین مورد توجه قرار گیرد. بر این اساس، مبحث حاضر مروری است برکار مونیکا کوهن و مشاوران کلینیک سرپایی caspar در کمبریج ماساچوست راجع به زنان مبتلا به سوء مصرف مواد که بر 25 سال تجربه جمعی و دانسته های دست اول و آخرین پژوهشهای مربوط به این موضوع متکی است (سال 2000). کلینیک فوق، برای افراد و خانواده هایی که درگیر یک یا چند نوع اعتیاد هستند، درمان جامع سوء مصرف مواد را تدارک می بیند.

Title: اختلال شدید زبان بیانی در کودک مبتلا به نابینایی مادرزادی از نوع لبر

http://rehabilitationj.uswr.ac.ir/article-1-541-fa.html

کودک 4 ساله مبتلا به نابینایی مادرزادی لبر، به دلیل مشکلات شدید گفتاری، به کلینیک گفتار درمانی ارجاع داده شد. در ارزیابی، تمایز قابل توجهی بین توانمندیهای زبان درکی و زبان بیانی به نفع زبان درکی مشاهده شد. اختلالات رفتاری نظیر لجبازی، وابستگی به اشیا و همچنین حرکات کلیشه ای در بیمار موجب ارجاع وی برای معاینات روانپزشکی شد که برای کوتاه مدت دارو تجویز شد. از آنجایی که مشخص شد کودک قادر به درک و تولید وزنهای مختلف است، از این توانمندی برای تربیت گفتار استفاده شد. این روش آموزشی، نتایج موفقیت آمیز زیر را داشته است: اختلالات رفتاری کودک کنترل شدند، کودک برای برقراری ارتباط شروع به استفاده از واژه ها و شبه واژه ها و شبه واژه ها کرد و کارکردهای شناختی وی رشد بهتری نشان دادند.
